# Investigation of Long-Term Roving Artisanal and Small-Scale Gold Mining Activities Using Time-Series Sentinel-1 and Global Surface Water Datasets

**DOI:** 10.3390/ijerph19095530

**Published:** 2022-05-02

**Authors:** Satomi Kimijima, Masayuki Sakakibara, Masahiko Nagai

**Affiliations:** 1 Research Institute for Humanity and Nature, Kyoto 603-8047, Japan; sakaki@chikyu.ac.jp; 2 Graduate School of Science & Engineering, Ehime University, Matsuyama 790-8577, Japan; 3 Graduate School of Science and Technology for Innovation, Yamaguchi University, Ube 755-8611, Japan; nagaim@yamaguchi-u.ac.jp; 4 Center for Research and Application of Satellite Remote Sensing, Yamaguchi University, Ube 755-8611, Japan

**Keywords:** active mining, alluvial mining, artisanal and small-scale gold mining, Indonesia, remote sensing, SAR, surface water occurrence

## Abstract

Artisanal and small-scale gold mining (ASGM) is a significant source of gold production globally despite the sector being informal and illegal. The rapid increase in the number of roving mining camps has negatively impacted the surrounding environment; however, the formation and transformation of roving mining camps have not been well studied. This study investigated the long-term trends and significant hotspots of roving camp-type ASGM (R-C-ASGM) in Katingain Regency, Central Kalimantan Province, Indonesia, from 1988 to 2020 using remotely sensed data, including Sentinel-1 time-series, global surface water (GSW), and world landcover datasets. Results show that several active R-C-ASGM sites existed in the Galangan and Kalanaman areas in 2017/2018. According to the GSW dataset, the Galangan area was estimated to be formed earlier, whereas the Kalanaman areas were recently formed and were associated with the Kalanaman river expansion. Notably, the center of Galangan was still a significant R-C-ASGM hotspot. The findings of this study broaden our understanding of R-C-ASGM transformation and identify significant R-C-ASGM hotspots over a long period. This study contributes to the development of timely and appropriate interventions for strengthening environmental governance.

## 1. Introduction

Artisanal and small-scale gold mining (ASGM) is a significant source of gold production globally. ASGM is the world’s largest employer in the gold mining sector, employing 70–80% of informal small-scale workers [1]. This has been carried out continuously in more than 80 countries as a tool for poverty alleviation and socioeconomic development [2,3]. In Indonesia, both active and inactive ASGM sites have been identified in 93 regencies in 30 of the country’s 34 provinces, with more than 1200 hotspots estimated in 2017 [4] and 250,000–300,000 miners [5]. Although this sector has provided economic benefits at various levels, the substantial harmful environmental and health risks associated with mercury pollution are devastating [6,7,8,9,10].

Kalimantan Island is an ASGM hotspots with alluvial operations [4]. Illegal mining activities are widespread on this island, even in conserved areas, negatively affecting biodiversity and human health [4]. Alluvial-based ASGM activities affect waterbodies and the surrounding environment, resulting in deforestation; high mercury contamination; and changes in geomorphological processes, biogeographic conditions, hydrological regime, and river courses [11,12].

The ASGM sector can be categorized into “travel-type” and “camp-type.” For the former, miners commute daily from their local residences to the mining sites. For the latter (hereafter called C-ASGM), miners live and conduct mining activities at informal worksites [13]. C-ASGM can be further categorized as either roving or non-roving practices. Both in terms of size and workforce, the ASGM sector has grown in tandem with increases in gold prices since 2000 [14]. Studies of C-ASGM in Indonesia have demonstrated the sector’s growth and the magnitude of its activities [13,15,16]. Accordingly, the sector’s rapid growth is anticipated to accelerate the environmental and health risks at various levels and on a wider scale.

As an adopter of the Minamata Convention on Mercury (MCM) initiated by the United Nations Environment Programme, Indonesia is attempting to reduce mercury use in the ASGM sector. The MCM is a global treaty that protects human health and the environment from anthropogenic emissions, mercury releases, and its compounds [17] and was adapted and implemented in October 2013 and August 2017, respectively [18]. The MCM’s Article 7 focuses primarily on the ASGM sector, facilitating the formalization of action plans and various regulations at a country level among the ratifying nations [19]. However, the policy formalization has often been hindered by insufficient institutional frameworks, capacities, and funds [20,21].

In addition to ASGM being informal, illegal, and unregulated, the uncontrollability of the sector by law allows for increased mercury use, endangering the environment and human health. Furthermore, geographical characteristics of the C-ASGM sector, for instance, the fact that most C-ASGM sites are located in remote rural areas, restrict the collection of information, such as the sector’s status and transformation, making it difficult to monitor them [13]. Remote sensing technologies enable the monitoring of time-series spatial changes in the C-ASGM sector to gain a better understanding of the sector. Satellite observations have been used to quantify the C-ASGM sector [13,15,16]. Cloud-free data sets are difficult to obtain in areas prone to heavy rainstorms. However, synthetic aperture radar (SAR), an active independent Earth observation system [22], can be a suitable alternative to measure optical data [23], facilitating the qualitative and comprehensive understanding of the C-ASGM sector.

Previous studies on the transformation of roving camp-type ASGM (R-C-ASGM) have investigated the active and inactive status of R-C-ASGM and its changes using Sentinel-1 (S-1) time-series datasets [16]. However, the availability of S-1 datasets is restricted to 2014. To investigate long-term R-C-ASGM practices, tracking surface water occurrence (SWO), which represents the frequency of land surface water from 1984 to 2020, associated with the spatial distributions of active R-C-ASGM sites may be key to recognizing the formation periods of mines in this sector. Therefore, this study primarily investigated a long-term trend and significant hotspots of R-C-ASGM from 1988 to 2020 in Katingan Regency, Central Kalimantan Province, Indonesia, using S-1 time series, global surface water (GSW), and world land cover datasets.

## 2. Materials and Methods

### 2.1. Overall Methodological Workflow

**Figure 1:**
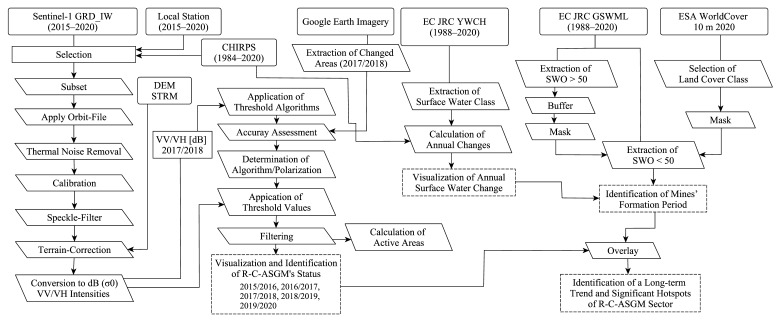
Overall methodology.

The methodological workflow used in this study is depicted in Figure 1. This workflow comprised four main steps to achieve its primary objective of investigating long-term R-C-ASGM trends and significant R-C-ASGM hotspots. First, the R-C-ASGM status from 2015 to 2020 was identified using the S-1 temporal time series. Second, the surface water extents observed from 1988 to 2020 were extracted. Third, targeted SWOs were extracted, along with a landcover map. Fourth, significant R-C-ASGM hotspots were identified by overlaying the results generated in steps 1 and 3. The results from these steps improve our understanding of the significant long-term trend of R-C-ASGM practices in the study site. This paper presents a discussion based on the findings described above. The methods employed in each step are explained in the following sections.

### 2.2. Study Area

**Figure 2:**
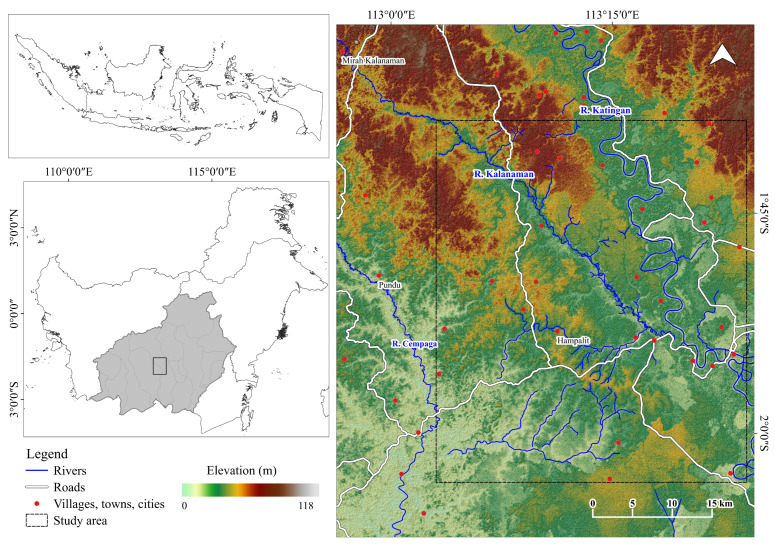
Study area.

Indonesia is a well-mineralized metallogenic region with significant gold mineralization associated with quartz veins in andesite-hosted epithermal settings. Gold-bearing alluvial soils in Central Kalimantan, a significant ASGM hotspot, have attracted several ASGM-targeted migrants from Java and South Kalimantan [24]. In this study, we focused on mining activities in Galangan, the center of Katingan Regency, Central Kalimantan Province, Indonesia, where the alluvial-based mining method is employed (Figure 2). In the early 1990s, the Galangan mining region rapidly developed and was designated as the geographical and historical center of land-based mining areas [25]. Especially, Hampalit town served as a base for mining activities for both indigenous miners and gold companies [24,26]. To date, migrated miners have continuously practiced R-C-ASGM to greater extents in various areas ranging from Kalanaman, Pundu, and Galangan to explore newer locations with more outstanding gold production by season [24]. Particularly, alluvial-based ASGM in this region employs various techniques, such as open pits, deep excavation pits, and floating pumps. For example, the open-pit method involves removing all soils and vegetation landscape from the surface, creating a barren wasteland [24,26]. Moreover, the floating pump method disturbs riverbanks and increases sediment volumes [24].

### 2.3. Identification of Active and Inactive Status of R-C-ASGM Sites

#### 2.3.1. S-1 Imagery

Six level-1 grand range detected (GRD) S-1 datasets covering 2015–2020 downloaded from the European Space Agency (ESA) were used to investigate the status of R-C-ASGM practice and its change. Through the EU/ESA Copernicus program, the S-1 mission (S-1A and S-1B) provides an exceptional combination of high spatial (10 m) and temporal (6 days) resolution data by operating two polar-orbiting radar imaging systems working with C-band (~5.7 cm wavelength). The main operational mode is the Interferometric Wide swath mode (IW) with VV/VH polarizations, which are freely and routinely available [27]. By referring to the Climate Hazards Group InfraRed Precipitation with Station (CHIRPS) and local station data, we used datasets acquired between July and August for the study period with a relative orbit number of 3 for a better comparison of the backscatter intensity of each image. Table 1 summarizes the main specifications of the databases used in this study.

#### 2.3.2. Image Preprocessing

The ESA’s open-source software, Sentinel Application Platform (version 8.0.0), was used for image preprocessing. The following five steps were implemented in the S-1 Toolbox: (1) orbit correction, (2) thermal noise removal, (3) radiometric calibration, (4) speckle filtering with 5 × 5 windows, and (5) terrain correction using the 3-arcsec digital elevation model (DEM) from the Shuttle Radar Topography Mission (SRTM) [28]. As a result, all imagery was converted to the digital pixel value of S-1 images, resulting in an image intensity value of σ^0^.

#### 2.3.3. Selection of Threshold and Detection of Changed Extents in Time Series

After image preprocessing, we identified optimized threshold values by the VV/VH polarizations acquired in 2017 and 2018. Sixteen different automatic global thresholding algorithms [29,30,31,32,33,34,35,36,37,38,39,40,41,42,43] were used in this process to identify mining-induced areas using an open-source Java image processing package, namely, Fiji (version 2.1.0) software (https://imagej.net/software/fiji/ accessed on 1 March 2022). Moreover, a supervised classification method, such as histogram intersection, was used. To determine the best separability for change detections, results were validated using high-resolution images obtained on 9 June 2017, and 23 September 2018, through Google Earth Pro. One hundred points were randomly selected from the datasets. Consequently, the best combination of algorithm and polarization was applied to all datasets. Next, the post-classification of a majority filter with a moving window size of 5 × 5 pixels was used to remove isolated pixels. After extracting the differences between the two target years, the areas observed in the river buffers were further eliminated to remove mudflats, possibly caused by the changes in the annual precipitation volume between the years. Consequently, the annual changes in the extent of illegal mining were computed for the following five temporal series: 2015/2016, 2016/2017, 2017/2018, 2018/2019, and 2019/2020. The correlation between detected active areas and the Indonesian gold price was evaluated statistically at the 95% confidence level.

### 2.4. Identification of SWO

In this process, European Commission (EC) Joint Research Centre (JRC) Yearly Water Classification History, v1.3 (YWCH, 1988–2020), EC JRC Global Surface Water Mapping Layers v1.3 (GSWML, 1988–2020), and ESA WorldCover 10 m 2020 (WC2020, 2000) datasets were used to determine a long-term change in surface water extent and its occurrence. The YWCH dataset contains yearly classifications of the seasonality of water detected throughout the year [44]. The GSWML dataset contains different facets of surface water data. Both datasets were generated on the basis of Landsat 5, 7, and 8 with 30 m ground resolution. Further, the WC2020 dataset provides a global land cover map of 2020 generated on the basis of S-1 and Sentinel-2 datasets with 10 m ground resolution.

First, seasonal and permanent water classes were extracted from the YWCH dataset, and long-term changes in surface water extents were identified. Trends of annual permanent and seasonal surface water were evaluated statistically using Sen’s Slope test with significance at the 95% confidence level. Meanwhile, the trend of monthly precipitation was calculated from 1985 to 2020 to validate the obtained results. Second, the occurrence band of the GSWML dataset, representing the frequency of water from 1984 to 2020, was primarily used to investigate specific surface water extents, which R-C-ASGM activities may have caused. Here, SWO greater and less than 50% (SWO > 50 and SWO < 50) were primarily considered permanent surface water and temporal- and mining-induced surface water extents, respectively. Third, a 50 m buffer was applied to SWO > 50 images. Fourth, the barren/sparse vegetation class among 11 land classes was extracted from the WC2020 dataset. Fifth, SWO < 50 images were masked by the buffered SWO > 50 and the barren/sparse vegetation class extracted from the WC2020 dataset. Here, buffering was applied to avoid possible errors, such as over-extraction of SWO < 50 at river edges, probably caused by changes in annual precipitation volume.

### 2.5. Identification of Long-Term Trends and Hotspots of R-C-ASGM

Long-term trends of R-C-ASGM and its hotspots were investigated by overlapping the results generated from Section 2.3 and Section 2.4.

## 3. Results

### 3.1. Determination of Threshold and Polarization Channels

Both VV/VH polarizations acquired in 2017 and 2018 were primarily used to derive the best combination of algorithm and polarization to identify illegal mining-induced landcover changes. Changed areas identified from each result were validated using features extracted from high-resolution Google Earth images, as mentioned in Section 2.5. Thus, this study found the best locally sensitive algorithm and polarization combination to be the IJ_Isodata algorithm and VH polarization, achieving 76.0% accuracy. Subsequently, the following image-specific thresholding values were generated for the final classification, probably leading to better results in detecting annual changes caused by active R-C-ASGM activities: −20.88 (2015), −19.95 (2016), −21.47 (2017), −20.16 (2018), −20.36 (2019), and −20.76 (2020).

### 3.2. Transformation of R-C-ASGM in Time Series

**Figure 3:**
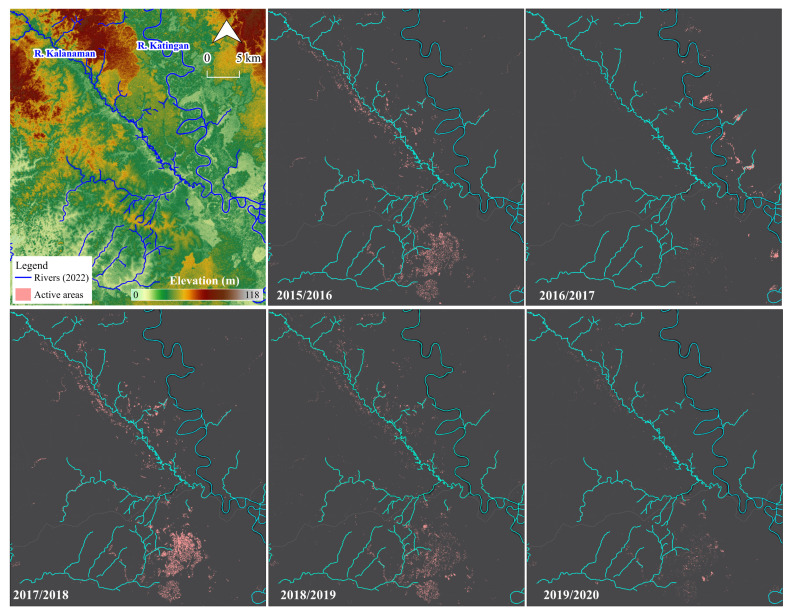
Transformation of active R-C-ASGM sites detected from IJ_Isoda algorithm and VH polarization.

The occurrences of active mining sites in the five periods (2015/2016, 2016/2017, 2017/2018, 2018/2019, and 2019/2020) are shown in Figure 3. The possible active mining areas were estimated to be 18.2 km^2^ (2015/2016), 6.5 km^2^ (2016/2017), 26.2 km^2^ (2017/2018), 14.5 km^2^ (2018/2019), and 4.8 km^2^ (2019/2020). The 2017/2018 period exhibited the peak change; meanwhile, smaller changes were observed in 2016/2017 and 2019/2020 in the study area. The detected areas were primarily located in the center of the Galangan region and the west of the Kalanaman River. After the implementation of the MSM in 2018, the mining activities in these detected areas decreased. Additionally, a negative correlation of −0.51 was found between the detected active areas and the Indonesian gold price from 2016 to 2020.

### 3.3. Surface Water Extents in Time Series

**Figure 4:**
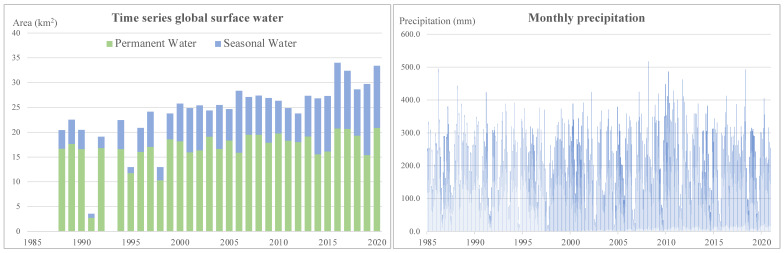
Trends of global surface water and monthly precipitation.

An increase in yearly surface water extent was found in the study area, from 20.4 km^2^ in 1998 to 33.4 km^2^ in 2020 (Figure 4). The analysis indicated that an increase in the amount of seasonal water was observed from 2000. According to the statistical test described in Section 2.4, positive increase trends were found in both permanent and seasonal water. Furthermore, a higher slope of 0.30 was found in seasonal water than in permanent water (0.12). In comparison, no trends were statistically identified from the monthly precipitation during 1985/2020 in the study area.

**Figure 5:**
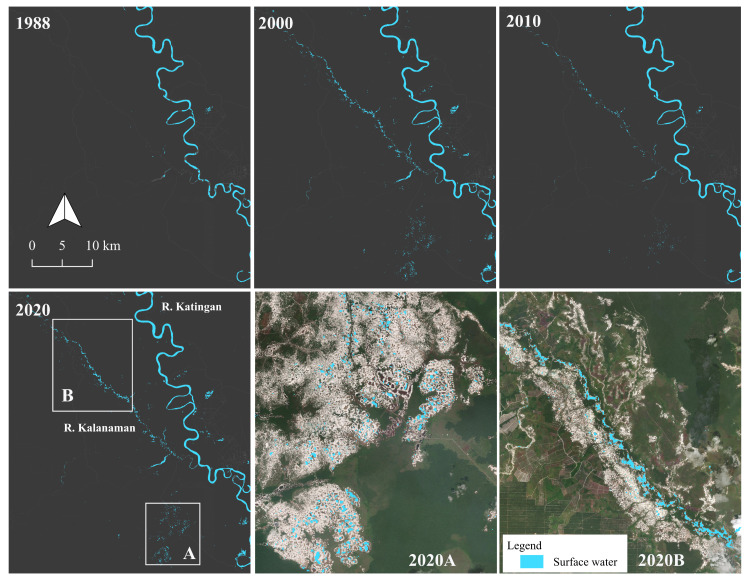
Changes in yearly surface water.

Changes in the yearly amount of surface water, particularly in 1988, 2000, 2010, and 2020, are shown in Figure 5. There were no changes in the Katingan River, whereas in the Kalanaman River, some changes were observed in 2000 and 2010, and the river’s extent expanded toward the northwestern parts of the Kalanaman area, forming the river (B in Figure 5). Moreover, surface water was also observed at the center of the Galangan area between 2000 and 2010 (A in Figure 5). This site is located further away from the main river networks but expanded its extent until 2020.

### 3.4. Surface Water Occurrence Associated with R-C-ASGM Activities

**Figure 6:**
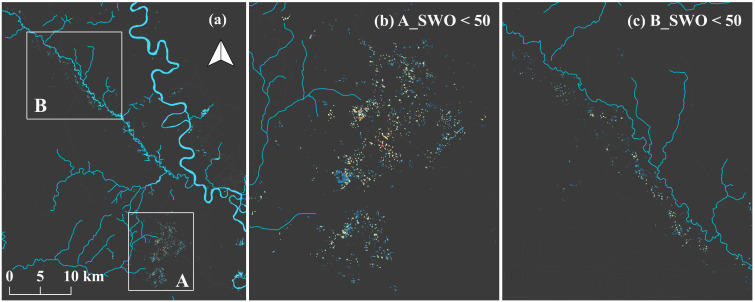

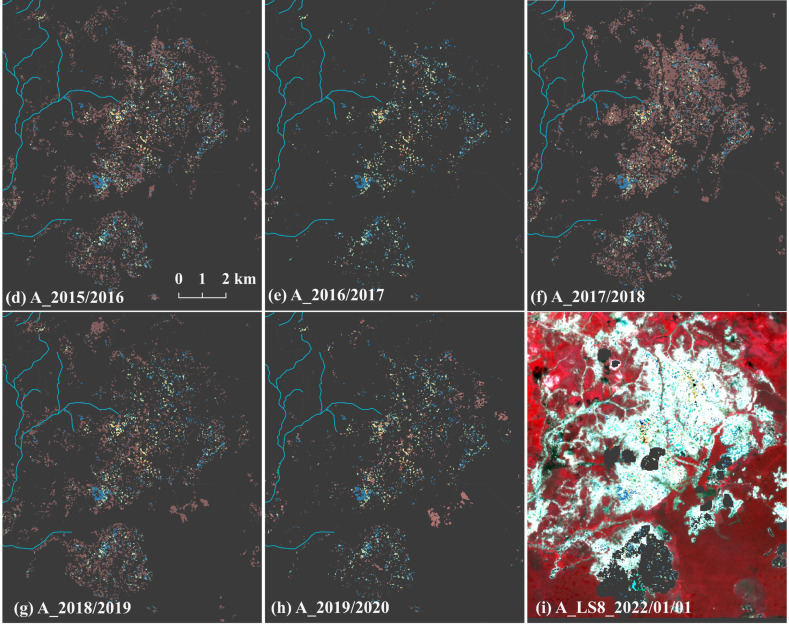

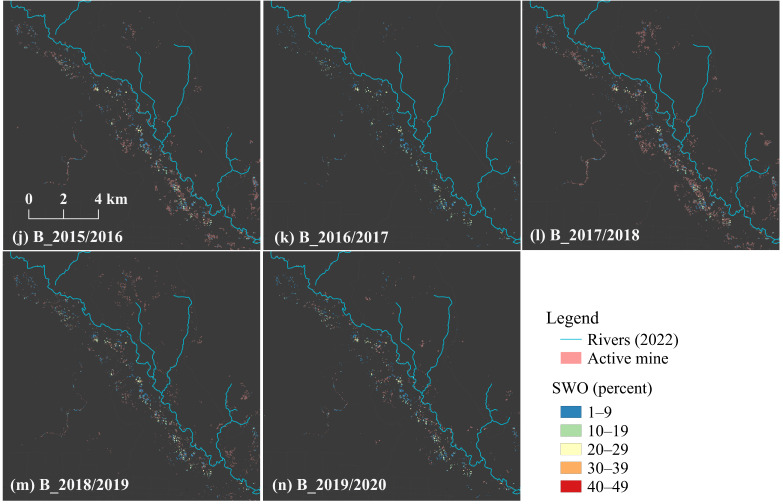
R-C-ASGM hotspots revealed by overlaying with the identified active mine sites and SWOs: (**a**) regional overview of SWO < 50 and waterbodies; (**b**) Galangan area (**A**); (**c**) Kalana-man area (**B**); (**d**–**h**) SWO < 50 and detected active sites in Galangan area in 2015/2016, 2016/2017, 2017/2018, 2018/2019, and 2019/2020, respectively; (**i**) overlaying SWO < 50 on Landsat8 imagery acquired on 1 January 2022; (**j**–**n**) SWO < 50 and detected active sites Kalanaman area in 2015/2016, 2016/2017, 2017/2018, 2018/2019, and 2019/2020, respectively.

The SWO < 50 observed during 1984–2020 was extracted, as described in Section 2.4, showing a total extent of 0.25 km^2^ in the study area. The SWOs in the Galangan area were as follows: 1–9% (75.1%), 10–19% (19.8%), 20–29% (4.1%), 30–39% (0.6%), and 40–49% (0.3%). Moreover, the SWOs in the Kalanaman area were as follows: 1–9% (64.8%), 10–19% (22.7%), 20–29% (9.1%), 30–39% (2.6%), and 40–49% (0.9%). Furthermore, the results were overlayed on the possible active R-C-ASGM sites found in Section 3.1 (Figure 6). Notably, a higher density of SWO was observed in the central of the Galangan area (A in Figure 6). In comparison, a lower density of SWO was observed in surrounding areas. Their distributions were toward the eastern, northern, and southern areas from the center of the Galangan area. Water areas separately identified in the southern part exhibited relatively lower SWOs. In comparison, relatively lower SWOs were observed along the Kalanaman River in the Kalanaman area (B in Figure 6). Slightly higher SWOs were found at approximately 3 km intervals along the river. Conversely, lower SWOs were found in the northern and southern parts of the river. Fewer pixels were observed, primarily in the river’s northern part. According to the overlay analysis, notably, possible hotspots of active mines were mostly observed in similar areas with SWO < 50 during the study period.

## 4. Discussion

### 4.1. Time-Series Analysis of R-C-ASGM

The time-series analysis contributed to revealing a pattern of R-C-ASGM activities, which helps predict future activity trends. According to the results, the most active period of R-C-ASGM during the study period occurred in 2017/2018. Despite being an informal sector, it is predicted that an increase in global gold prices accelerates a massive entry of immigrants from different islands into mining sites. The gold price in Indonesia has risen since 2007 (5,722,115 IDR/oz. in January), with an especially steady rise since 2017 (15,907,804 IDR/oz. in January), with the price nearly doubling by 2020 (26,257,748 IDR/oz. in December) [45]. Previous studies have indicated a strong relationship between the development of the C-ASGM sector and increases in gold prices [13,15]; however, this study shows a moderate negative correlation. This trend may be attributed to the coronavirus pandemic affecting mining activities and the gold market rather than the adaptation of the MCM. Although the gold market price increased during the pandemic, mining operation costs increased because of the disruption in labor, supply chains, and cash flow, increasing the gold price [46,47].

### 4.2. Tracing Mines’ Formation Period and Hotspots in R-C-ASGM

While GSW datasets generated from the Landsat series may have weather-related effects, the SWO datasets can be a significant indicator of transformations of R-C-ASGM activities at a regional level. A long-term quantitative analysis of R-C-ASGM broadens our understanding of the scale and pattern of their transformation over time, as well as tracking its responses to global factors, such as the MCM and the gold price. Furthermore, estimating the formation period of mines and recognizing significant R-C-ASGM hotspots is essential to identify a significant source of high pollution, which may lead to significant socio-environmental destruction at the local and community levels.

The results of this study quantified significant SWOs resulting from R-C-ASGM activities and demonstrated their hotspots, along with the status of R-C-ASGM mines. Most notably, in 2017/2018, an active area peak was identified, which was largely concentrated at the center of the Galangan area and along the Kalanaman River. Similarly, higher SWOs were observed in the central of the Galangan area. Conversely, lower SWOs were observed in their surrounding areas and along the Kalanaman River. This trend may indicate that the central Galanga area was formed earlier and is still a significant hotspot. However, the Kalanaman area was recently formed, expanding toward the northeast along the river. The alluvial-based R-C-ASGM practices have accelerated fluvial changes with this expansion [11]. To date, only a few studies have quantified the transformation of the R-C-ASGM sector using remote technology. The application of SAR technology enables the monitoring of R-C-ASGM changes. Previously, [16] investigated the active and inactive status of R-C-ASGM practices from 2015 to 2021 using the S-1 temporal series. Conversely, our work quantified the transformation of R-C-ASGM practice for a longer time frame while also using global surface datasets. The utilization of SWO associated with the spatial distributions of active R-C-ASGM helps in estimating the formation periods of R-C-ASGM mines. Furthermore, overlaying the R-C-ASGM status contributes to detecting mining hotspots, which may be a significant source of high pollution, leading to the destruction of the surrounding environment and increasing health risks.

### 4.3. Limitations

The results of this study have some limitations in terms of the quality of input data. First, precipitations that occurred before the acquisition time can decrease the backscatter intensity in polarizations, overestimating illegal mining extents. Second, some smaller areas were undetected because of the spatial resolution of the datasets used.

## 5. Conclusions

In this study, the long-term trend and significant hotspots of R-C-ASGM in Katingan Regency, Central Kalimantan Province, were investigated using time-series S-1, YWCH, GSWML, and WC2020 datasets. The results show a massive occurrence of active R-C-ASGM sites with 2017/2018 as the peak period, primarily at the center of the Galangan area and along the Kalanaman River. With the combination of SWO datasets, the Galangan area was estimated to have formed earlier than other study areas, and its central area was still a significant hotspot. Conversely, Kalanaman areas were recently formed, and their sites expanded with the creation of the Kalanaman river. Therefore, the long-term trend of R-C-ASGM and its significant hotspots can be detected from a combination of time-series datasets. These quantitative analysis results broaden our understanding of R-C-ASGM distributions, transformation, mine occurrence periods, and significant hotspots over a long period. Recognizing long-term R-C-ASGM transformation and identifying significant R-C-ASGM hotspots are also essential to tracking R-C-ASGM responses to global factors/events, such as the MCM and gold prices. This further helps predict the magnitude of environmental destruction at the local and regional levels. These findings are expected to assist in developing rapid and appropriate interventions for strengthening environmental governance by involving various stakeholders.

## Figures and Tables

**Table 1:** Main specification of satellite imagery used in this study.

Satellite	Type	Acquisition Date	Spatial Resolution	Image Number	Polarization	Wavelength
Sentinel-1	C-SAR	20 July 2015	10 m	3	Descending (VV, VH)	C band
		7 August 2016				
		21 July 2017				
		4 July 2018				
		11 July 2019				
		10 August 2020				

## Data Availability

Not applicable.
